# Artificial intelligence in experimental and clinical in vitro analysis: applications, limitations, and future directions

**DOI:** 10.1016/j.biotno.2026.08.003

**Published:** 2026-09-11

**Authors:** Zhinya Kawa Othman, Mohamed Mustaf Ahmed, Rahmatullah Nazari, Adamu Muhammad Ibrahim

**Affiliations:** a Faculty of Pharmaceutical Sciences, Chulalongkorn University, Bangkok, Thailand; b Faculty of Medicine and Health Sciences, SIMAD University, Mogadishu, Somalia; c Faculty of Dentistry, Cheragh Medical University, Kabul, Afghanistan; d Department of Biomedical Science and Biomedical Engineering, Faculty of Medicine, Prince of Songkhla University, Hat Yai, Thailand

**Keywords:** Artificial intelligence, In vitro analysis, Machine learning, Laboratory automation, Deep learning, High-throughput screening

## Abstract

The integration of artificial intelligence into in vitro analyses represents a transformative shift in laboratory medicine, diagnostics, and pharmaceutical research. Traditional in vitro methods, from cell culture assays to high-throughput screening, rely on manual interpretation and conventional statistics. Machine and deep learning now automate image analysis, improve diagnostic accuracy, accelerate drug discovery, and enhance quality control. Convolutional neural networks and predictive models have reported specialist-level performance in cell segmentation, biomarker detection, and pathological classification, although mainly in internal benchmark studies rather than in prospective clinical validations. However, important limitations persist, including dependence on data quality and standardization, limited external and prospective validation, algorithmic transparency, ethics and data governance, regulation, and unequal access to computing infrastructure. We examine the applications, limitations, and future directions of this technology. In this review, in vitro analysis refers to the laboratory examination of cells, tissues, and biological samples outside the living organism, and it spans both experimental discovery research and clinical in vitro diagnostics, which share analytical challenges but differ in their validation and regulatory requirements.

## 1 Introduction

In vitro analysis underpins modern diagnostic medicine, pharmaceutical development, and biomedical research, with clinical laboratory testing widely recognized as a central input to medical decision-making.^1^^,^^2^ These techniques span routine hematology and clinical chemistry, microbial culture, immunoassays, histopathology, and complex multiparameter drug screening platforms. Despite their centrality, laboratory workflows have long contended with errors that arise across the pre-analytical, analytical, and post-analytical phases; most mistakes occur before (46–68.2% of total errors) and after (18.5–47%) the analytical step rather than during analysis itself.^2^ Traditional approaches to cell counting, morphological assessment, and biochemical quantification rely heavily on trained personnel, whose performance can vary based on experience, fatigue, and subjective interpretation. These constraints have prompted growing interest in automation and computational assistance across the clinical laboratory.^3^

Artificial intelligence (AI), defined as computational systems capable of performing tasks that ordinarily require human cognition, has been increasingly proposed as a means of addressing several of these workflow challenges.^3^ Machine learning (ML), a subset of AI, allows algorithms to learn statistical patterns from data without explicit task-specific programming,^4^ while deep learning uses multi-layered neural networks to extract hierarchical representations from complex inputs.^5^ The combination of large clinical datasets and modern computing power has driven a wave of clinical AI applications across health care.^6^^,^^7^ High-profile demonstrations include skin-lesion classification at dermatologist-level accuracy,^8^ screening mammography evaluated across multiple international cohorts,^9^ and the broader convergence of human and AI in clinical practice.^7^ These technologies have likewise been deployed across clinical and genomic diagnostics, including applications in radiology, dermatology, and genomics,^10^ and their application to in vitro laboratory analysis is expanding alongside these adjacent diagnostic domains.^3^

Regulatory activity reflects the rapid translation of AI tools into clinical practice. A taxonomy of 1016 United States Food and Drug Administration (FDA) authorizations for AI- and ML-enabled medical devices issued between 1995 and 2024 identified 736 unique devices, of which 84.4% used images as their principal input.^11^ Radiology was the lead review panel for 88.2% of these imaging devices, whereas hematology accounted for 1.9%, and only five devices in total were based on omics data, indicating that in vitro diagnostic and laboratory-medicine applications remain a small minority.^11^ However, its adoption in laboratory medicine remains uneven. In a survey of 128 laboratory medicine stakeholders, only 15.6% reported that AI was already in use in their organization; respondents identified high investment costs, unproven clinical benefit, and privacy concerns as the principal barriers, and education, workflow integration, and evidence of clinical utility as the measures needed to mainstream AI in the field.^12^

A systematic review and meta-analysis of AI in digital pathology covered 100 studies and more than 152,000 whole-slide images and pooled 48 of them, yielding a mean sensitivity of 96.3% and a mean specificity of 93.3%; the authors cautioned that these estimates should be interpreted with care given the heterogeneity of study designs.^13^ A separate systematic review of deep learning in medical imaging covered 82 studies and pooled the 14 that provided direct head-to-head comparison with healthcare professionals, reporting a sensitivity of 87.0% and a specificity of 92.5% for the deep learning models against 86.4% and 90.5% for the professionals assessed on the same samples, although that comparison was restricted to the contingency table reporting the highest accuracy in each study.^14^ In cellular image analysis specifically, large-scale annotated tissue datasets and dedicated deep learning architectures have likewise been reported to achieve human-level segmentation performance across diverse tissues and imaging platforms.^15^ Collectively, these syntheses converge to provide a consistent picture: across digital pathology, medical imaging, and cellular image analysis, aggregate AI accuracy approaches that of trained human readers, yet in every case the pooled estimates rest on retrospective, heterogeneous, and largely internally validated datasets, and they therefore characterize the potential of AI for experimental and clinical in vitro analysis rather than its demonstrated performance in routine practice.^13^, ^14^, ^15^

For the purposes of this review, in vitro analysis denotes the laboratory examination of cells, tissues, microorganisms, and other biological samples outside an intact organism. This definition encompasses two related, but distinct, domains. The first is experimental and discovery research, including cell culture, microscopy, high-content and high-throughput screening, and drug discovery research. The second is clinical in vitro diagnostics, including the analysis of patient-derived blood, tissue, microbial, biochemical and histopathological samples. Both domains share challenges of standardization, reproducibility, data quality, validation, and interpretability; however, they differ in emphasis: discovery research prioritizes scalability, throughput, and model optimization, whereas clinical diagnostics must contend with limited and sensitive patient samples, pre-analytical variability, clinical validation, regulatory compliance, and direct consequences for patient care. To keep this scope tractable, the present review concentrates on cell- and tissue-based in vitro analysis and is organized such that discovery research and clinical diagnostic applications are treated as distinct, clearly identified contexts throughout. We aim to review the current applications, efficiency gains, and future directions of AI in in vitro analysis, with particular attention to the evidence supporting its integration into laboratory workflows. Throughout, experimental discovery research is distinguished from clinical diagnostic applications, and the reported benefits of each application are weighed against its limitations and validation requirements.

## 2 Historical evolution of in vitro analysis and the emergence of AI

The role of AI in the laboratory is best understood against the longer arc of laboratory practice. The history of in vitro analysis extends from the earliest microscopic examinations of blood and tissue specimens to the sophisticated automated platforms of the present day. Early laboratory medicine relied largely on manual techniques, with skilled technicians performing visual assessments of stained preparations using light microscopy. The progressive introduction of automated analyzers from the mid-twentieth century onward shifted parts of the field toward mechanization, enabling higher throughput and improved reproducibility across clinical laboratory disciplines.^16^^,^^17^

The digitization of laboratory data in the late twentieth century created the foundation upon which AI-based approaches were later built. Early computational applications in this field were largely rule-based, relying on predefined logic that limited their ability to adapt to heterogeneous patient populations and assay conditions. The subsequent deep learning revolution, catalyzed by the success of convolutional neural networks (CNNs) in image recognition around 2012, opened new possibilities for laboratory applications.^18^ These architectures are particularly well-suited for the analysis of microscopy images, histopathological slides, and other visual data types that are central to in vitro analysis. Since then, the field has expanded rapidly, with AI systems being applied across the full spectrum of laboratory workflows, from specimen preprocessing to result reporting.

## 3 ML approaches in microscopy and cell image analysis

Microscopy is among the most fundamental tools in in vitro analysis, and it is where the manual burden described above is most acute, as operators must identify, count, and classify cells or cellular structures one field at a time. ML algorithms, particularly CNNs, have been applied to automate these tasks and can render previously laborious cellular image analyses routine, with individual benchmark studies reporting performance comparable to that of expert annotators.^18^

Two comprehensive surveys of deep learning in microscopy image analysis reached convergent conclusions. Both document neural network-based methods for image classification and region segmentation across a range of microscopy modalities, and both report task-specific improvements over conventional image-processing approaches.^19^^,^^20^ The earlier survey additionally covers object detection,^20^ while the more recent one extends to object tracking and super-resolution reconstruction and identifies the acquisition and evaluation of training datasets as the principal outstanding difficulty.^19^

The democratization of deep learning tools, exemplified by platforms such as ZeroCostDL4Mic, has further accelerated adoption by lowering the technical barriers to implementation.^21^ These accessible frameworks allow researchers without extensive computational expertise to train and deploy custom models for specific microscopy applications, broadening the reach of AI-enhanced image analysis across diverse laboratory settings. These gains in throughput and reproducibility must be weighed against the important limitations. Performance depends heavily on annotation quality and can degrade when imaging platforms, magnifications, or staining protocols differ from training data. As a result, models developed in one laboratory often transfer poorly to another without retraining or domain adaptation.^22^^,^^23^

## 4 Deep learning for automated cell segmentation and classification

Among these microscopy tasks, segmentation has received the most attention. Cell segmentation, the process of delineating individual cells within a microscopy image, is a critical step in many in vitro analysis workflows. Traditional segmentation methods rely on thresholding, edge detection, and watershed algorithms, which often struggle with overlapping cells, variable staining intensities, and complex tissue architectures. Deep learning-based segmentation approaches have largely overcome these limitations by learning directly from annotated training data.^24^

Cellpose, a generalist deep learning algorithm trained on over 70,000 segmented objects, demonstrated that a single model could accurately segment cells across a wide range of image types without requiring retraining or parameter adjustments.^25^ The DeLTA framework showed that two consecutive U-Net models could integrate cell segmentation, tracking, and lineage reconstruction in a unified pipeline for time-lapse microscopy of *Escherichia coli* in mother-machine microfluidic devices, enabling automated analysis of dynamic cellular processes.^26^

DeepBacs extended these capabilities to bacterial image analysis, demonstrating multi-task deep learning approaches for segmenting brightfield and fluorescence images of different bacterial species.^27^ Test-time augmentation strategies have also been shown to improve the robustness of deep learning segmentation models by aggregating predictions across multiple transformed versions of the input image, with averaging used for semantic segmentation models and pixel-level majority voting used for instance segmentation models.^28^ Taken together, these tools trace a common trajectory in the segmentation literature: models tuned to a single organism or imaging setup have progressively given way to generalist models and to model-agnostic strategies such as test-time augmentation, each step trading a degree of specialized accuracy for broader applicability.^25^, ^26^, ^27^, ^28^ Despite these advances, segmentation performance remains dependent on how closely deployment images resemble the training data, and domain shift between laboratories measurably alters model behavior in histopathology^22^; reported metrics are also typically obtained on curated benchmark datasets that may overstate performance in routine clinical use.^29^ The selected deep learning architectures and their reported performance characteristics for cell image analysis are summarized in Table 1.

**Table 1:** Selected deep learning architectures and their performance in cell image analysis.

Method (application domain)	Input data	Dataset and validation design	Quantitative performance	Principal limitation	Level of implementation	Source
DeepBacs (multi-task bacterial imaging)	Brightfield and fluorescence bacterial images	Multiple bacterial species; open-source multi-task benchmarks; internal evaluation	Open-source multi-task segmentation across species	Species- and imaging-specific; requires annotated training data	Research, open-source tool	27
Cellpose (generalist cell segmentation)	Diverse light-microscopy cell images	More than 70,000 manually segmented objects; internal test on varied image types	Accurate segmentation across image types without retraining	Generalist model; may need fine-tuning for atypical morphologies	Research tool, widely adopted	25
ZeroCostDL4Mic (accessible deep learning)	User-provided microscopy images	User-defined datasets; no standardized external validation	No-code model training and deployment	Quantitative evaluation tools are provided, but training-data quality and external validation remain the user's responsibility	Research platform	21
DeLTA (segmentation, tracking, lineage)	Time-lapse microscopy of *E. coli* in microfluidic devices	Task-specific dataset; internal validation	Automated lineage reconstruction in a unified pipeline	Tailored to bacterial mother-machine setups; limited generality	Research method	26
Test-time augmentation (nuclei segmentation)	Nuclei microscopy images (2018 Data Science Bowl)	2018 Data Science Bowl challenge data; internal evaluation	Improved robustness of predictions	Adds inference cost; gains shown on benchmark data	Research method	28
Deep learning with watershed post-processing (2-D whole-cell segmentation)	Single-channel microscopy images of cell cultures stained with whole-cell markers	Images acquired with several markers and magnifications; internal validation	About 86% overlap with ground-truth segmentation	Benchmark-based; performance varies with cell type and imaging	Research method	24

## 5 AI in high-throughput screening and drug discovery

The same image-analysis capabilities support a second discovery-research application, one in which the unit of analysis is not the individual cell but the compound library. High-throughput screening (HTS) is a cornerstone of modern pharmaceutical development and the principal route to identifying starting chemical matter for a new drug, using biochemical and cell-based assay formats.^30^ Traditional HTS workflows generate enormous volumes of data that require sophisticated analytical pipelines. Beyond supporting these analyses, AI-based methods have also been proposed as computational alternatives to physical HTS, with structure-based deep learning models evaluating chemical libraries before compounds are synthesized and at potentially lower cost than experimental assays.^31^

A large industry-led study reporting on 318 prospective drug discovery projects, comprising 22 internal projects and 296 academic collaborations, demonstrated that the AtomNet CNN could identify active compounds with novel scaffolds across every major therapeutic area and protein class, even for targets lacking known binders or high-quality X-ray crystal structures.^31^ AI-accelerated high-throughput microfluidic systems have also been described for biomedical screening, in which ML is used to interpret the large data volumes such platforms generate and to support automated system control in drug screening.^32^

High-content imaging is a natural target for AI because these assays generate rich multidimensional datasets that simultaneously capture cellular morphology, protein expression, and subcellular localization. Deep learning algorithms can extract complex phenotypic features from such images, complementing the simpler readouts typical of single-parameter assays and supporting more detailed compound characterization.^33^ Such image-based profiling approaches are increasingly being used alongside traditional reporter assays in drug screening campaigns. Across both computational screening and image-based phenotypic profiling, the reported advantage is the same, namely, the broader coverage of chemical and phenotypic space than physical assays alone can achieve, and so is the reported constraint.^31^^,^^33^ Predictions depend on the quality and coverage of the training libraries and still require experimental confirmation: in the largest prospective virtual screening campaign reported to date, hit rates averaged 6.7% for internal projects and 7.6% for academic collaborations, so strong in silico performance does not by itself guarantee validated hits.^31^

## 6 ML-enhanced biomarker detection and biosensor integration

The applications considered so far belong to discovery research, and the same multivariate methods are also applied on the clinical side of in vitro analysis. Biomarker detection is central to in vitro diagnostics, and ML approaches have been increasingly applied to biomarker-based assays in recent years. By learning patterns across multi-analyte datasets, ML models can in principle identify biomarker signatures that are more informative than individual markers analyzed in isolation, although reviews of the field have also cautioned that such gains depend on careful study design to avoid overfitting and generalization failure.^34^

The integration of ML with advanced biosensor platforms has enabled the real-time multiplexed quantification of biomarkers. ML classification algorithms combined with immunoassays based on surface-enhanced Raman spectroscopy (SERS) in microfluidic chips have demonstrated enhanced specificity for cancer biomarker detection.^35^ More broadly, the convergence of ML with electrochemical, wearable, and fluorescence biosensors has been described as expanding the possibilities for biosensor-based detection, analysis, and diagnosis.^36^

Metabolomic profiling combined with ML has shown promise for early disease diagnosis, with a recent study demonstrating that metabolomic ML predictors could diagnose gastric cancer with high accuracy in an independent validation cohort.^37^ These approaches exemplify a broader trend toward multivariate analytical frameworks in which AI systems synthesize information from multiple biomarkers to generate diagnostic predictions that can outperform any single analyte.^36^^,^^37^ Across the immunoassay, biosensor, and metabolomic approaches considered above, the recurring finding is the same: multivariate ML models extract more diagnostic information than any single analyte.^35^, ^36^, ^37^ The recurring caveat is also the same. ML applied to high-dimensional biomarker data has a tendency to overfit, so a result obtained in a small group of samples may not generalize to wider patient populations because of the high likelihood of false discovery, and independent validation therefore remains necessary before clinical utility can be claimed.^34^

## 7 AI-driven laboratory automation and workflow optimization

Beyond analytical accuracy, AI has demonstrated substantial potential for optimizing laboratory workflows and reducing operational inefficiencies.^38^ Total laboratory automation (TLA) systems, which integrate specimen transport, processing, and analysis across the pre-analytical, analytical, and post-analytical phases, have begun to incorporate AI, ML, robotics, and Internet-of-Things technologies that support predictive analytics, quality control (QC), and automated data management.^38^

Studies evaluating the impact of AI-enhanced automation have reported substantial efficiency improvements in various fields.^39^^,^^40^ In a study applying deep learning to flow cytometric detection of minimal residual disease in chronic lymphocytic leukemia, AI-assisted gating reduced the analysis time per case from an estimated 15 min to approximately 12 s, while maintaining a correlation above 0.999 with expert analysis.^39^ AI tools have also been described as automating time-consuming pathology tasks and supporting more rapid diagnostic workflows.^40^ Many of these reported efficiency gains, however, derive from single-site studies, and the evaluation of medical AI more broadly shows the same pattern: in an analysis of 130 FDA-approved medical AI devices, 126 had undergone only retrospective evaluation at submission and 93 had no publicly reported multi-site assessment.^41^ The selected efficiency gains across laboratory workflow applications are summarized in Table 2.

**Table 2:** Quantitative efficiency gains from AI integration in laboratory workflow applications.

Method (application domain)	Input data	Dataset and validation design	Quantitative performance	Principal limitation	Level of implementation	Source
Flow cytometry gating (minimal residual disease, chronic lymphocytic leukemia)	Clinical flow cytometry data	Single-center study; comparison against expert analysis	Time per case reduced from about 15 min to about 12 s; correlation above 0.999 with experts	Single-center; not prospectively validated across sites	Clinical study, decision support	39
Pathology reporting (diagnostic pathology)	Whole-slide images	Narrative review evidence	Faster AI-assisted analysis versus manual review	Qualitative; magnitude varies by task and setting	Emerging clinical use	40
Medical imaging (deep learning versus clinicians)	Medical images (meta-analysis)	Systematic review; 14 head-to-head studies	Pooled sensitivity 87.0%, specificity 92.5%, versus 86.4% and 90.5% for clinicians on the same samples	Heterogeneous studies; few prospective or external	Evidence synthesis	14
Drug screening (AtomNet CNN)	Chemical structures and protein targets	318 prospective projects (industry-led)	Identification of active compounds with novel scaffolds; average hit rates 6.7% (internal) and 7.6% (academic)	Requires experimental confirmation; hit rates vary	Research and industry	31
Autonomous synthesis (robotic chemist)	Reaction and experimental parameters	Proof-of-concept implementation	Autonomous synthesis with minimal human input	Early stage; narrow scope; scalability unproven	Research demonstration	42

Several of these capabilities have been evaluated in real-world laboratory and clinical settings, rather than in controlled benchmarks alone. In a large clinical-grade study, a weakly supervised deep learning system trained on more than 44,000 whole-slide images from a single academic pathology department classified prostate cancer, basal cell carcinoma, and breast cancer metastases to axillary lymph nodes with high accuracy, including an area under the curve above 0.98 for prostate cancer detection, illustrating deployment at institutional scale.^43^ Independent assessment of the FDA-authorized Paige Prostate system reported strong standalone diagnostic accuracy for prostate cancer on biopsy material, with high sensitivity and specificity, while noting that generalization to other laboratories and scanners still requires ongoing validation.^44^ In hematology, multicenter evaluation of an artificial-intelligence-based peripheral blood smear platform reported high agreement with manual microscopy for the white-cell differential and red-cell morphology.^45^ These three evaluations differed in terms of specimen type, scale, and design: a single-institution deployment of a weakly supervised model across more than 44,000 slides, an independent single-center assessment of a commercial FDA-authorized algorithm, and a multicenter comparison of an automated smear platform against manual microscopy.^43^^,^^45^ Nonetheless, they converge on the same conclusion. AI-driven in vitro analysis is beginning to move from retrospective benchmarks to supervised clinical use, while real-world performance remains contingent on local validation.

The concept of self-driving laboratories, in which AI systems autonomously design, execute, and interpret experiments, represents the most advanced vision for laboratory automation. Recent implementations have demonstrated that AI-driven robotic platforms can autonomously synthesize organic molecules and optimize reaction conditions with minimal human intervention.^42^ These systems exemplify the convergence of robotics, ML, and laboratory science, which may define the next generation of in vitro analysis platforms.

## 8 QC and error reduction through intelligent systems

Throughput gains are significant only if the results are reliable. QC is a critical component of laboratory operations, and AI-based approaches have been investigated to improve the detection and prevention of analytical errors. In clinical chemistry, patient-based real-time quality control (PBRTQC) uses patient result patterns to monitor assay performance, and ML models, including regression-adjusted algorithms, neural networks, and anomaly-detection methods, have been proposed as next-generation alternatives to conventional moving-average approaches.^46^ One such model, traceable to certified reference data, reduced the average number of patient samples required to detect a critical bias from approximately 600 to 20 compared with established PBRTQC algorithms.^47^ Critical head-to-head comparison of these AI-based methods against conventional statistical QC has nonetheless only recently begun, and their principal drawbacks remain complexity and computational demand.^46^ In the related but distinct context of biomanufacturing, ML approaches have been applied to implement quality-by-design frameworks, supporting the modeling and prediction of critical quality attributes during the production of biologics.^48^

In the related field of biofabrication, ML has been proposed as a tool for QC across pre-process, in-process, and post-process stages of bioprinting workflows, enabling more consistent assessment of printed tissue constructs and facilitating the identification of structural defects that may compromise biological function.^49^

## 9 Regulatory landscape and clinical validation of AI-based in vitro tools

Whether any of these capabilities will reach patients depends on validation and regulatory approval. The clinical validation of AI systems in laboratory settings presents distinctive methodological challenges. Unlike static medical devices, AI algorithms may evolve through updates and retraining, raising questions about how regulatory approval should account for post-market algorithmic changes.

Many of the tools discussed in this review are currently used for research rather than patient care, and their transition toward clinical application is gated by regulatory approval. In the United States, an AI system intended for clinical in vitro diagnostic use must undergo analytical and clinical validation and obtain marketing authorization through the 510(k), De Novo, or premarket approval pathways before it can inform diagnosis or treatment.^50^^,^^51^ Requirements have recently been raised in the European Union, where the In Vitro Diagnostic Regulation, applicable since May 2022, reclassifies most digital pathology software from the self-certified general in vitro diagnostic class of the superseded In Vitro Diagnostic Directive to higher-risk Class C, which requires evidence review by a conformity assessment body.^52^ That change is still working through: under the earlier Directive, 24 of 26 AI-based digital pathology products on the European Economic Area and Great Britain markets had been self-certified as general in vitro diagnostic devices, only 38% had a peer-reviewed internal validation study and 42% a peer-reviewed external one, and Directive-compliant devices may remain on the market until May 2027 under transitional arrangements.^52^ A research-use-only algorithm that performs well on retrospective data does not automatically qualify for clinical use. Regulatory authorization and a life-cycle management approach built on recurrent local post-market performance monitoring, together with the flexible mechanisms regulators will need in order to keep pace with model change, are the routes through which laboratory AI is expected to move from discovery into supervised clinical use.^50^

Validation studies must demonstrate analytical accuracy and clinical utility across diverse patient populations. As noted above, the digital pathology meta-analysis that encompassed more than 152,000 whole-slide images reported a mean sensitivity of 96.3% and a mean specificity of 93.3% but also found that 99% of the included studies had at least one domain at high or unclear risk of bias or applicability concern.^13^ This finding underscores the need for a more rigorous study design as the field matures.

However, interpreting these performance figures requires caution. High technical performance, such as accurate segmentation or rapid image processing, does not establish biological relevance, diagnostic utility, or improved patient outcomes. It is therefore useful to distinguish technical performance from analytical validity, meaning whether an assay measures its target accurately and reproducibly; clinical validity, meaning whether the measurement predicts a clinically meaningful state; and clinical utility, meaning whether its use improves patient outcomes.^51^^,^^53^ A further consideration specific to in vitro models is biological relevance, that is, whether the measured feature reflects the biology of interest and is qualified for a defined context of use.^54^ Descriptions such as specialist-level, human-level, or performance comparable to experts, should be interpreted in this light. Their weight depends on whether they derive from internal benchmarks, external datasets, prospective studies, or real-world implementation; in most of the studies reviewed here, such claims arise from internal or benchmark comparisons rather than from prospective clinical evaluation, a gap that is not confined to the laboratory: none of the 54 high-risk devices in the FDA analysis cited above had been evaluated prospectively.^41^

## 10 Challenges and limitations of AI adoption in laboratory settings

Despite promising evidence for the use of AI in in vitro analyses, several substantial challenges hinder its widespread adoption. Data quality and standardization remain fundamental concerns, as ML models can be sensitive to dataset shift arising from technical differences between sites, including variation in equipment, coding definitions, electronic health record systems, and laboratory assays.^29^^,^^55^ Algorithmic transparency, often referred to as the black box problem, presents another barrier to clinical adoption. Many high-performing deep learning models lack interpretable decision-making processes, which may limit clinician trust and complicate regulatory evaluation.^56^ Infrastructure requirements, including computational hardware, data storage, network connectivity, and specialized technical personnel, may also be prohibitive for resource-limited laboratories, particularly in low- and middle-income countries.^57^

A related and often underappreciated constraint is the limited availability of large, curated, representative, and independently validated clinical laboratory dataset. Many published algorithms are trained on single-center, retrospective data, which limits their generalizability to new populations, instruments, and workflows.^29^^,^^55^ The consequences are measurable: in a systematic review of 572 ML studies predicting deterioration in intensive care, only 14.7% had been externally validated, and among those the area under the receiver operating characteristic curve fell by an average of 0.037 on external data, with a reduction greater than 0.05 in almost half of the studies.^58^ Robust translation therefore depends on multicenter datasets, harmonized metadata, clinically meaningful reference standards, and both prospective and external validation,^41^^,^^58^ together with recurrent local post-market performance monitoring for the dataset shift that follows deployment.^50^^,^^55^ Federated learning may ease data-access barriers, but it does not by itself resolve differences in data quality, annotation, measurement platforms, or patient populations.^59^

The ethical considerations raised by AI in in vitro analysis extend beyond data privacy and algorithmic bias. Reusing patient-derived samples and their associated clinical data for algorithm development raises questions of informed consent and secondary use,^60^ and even nominally anonymized datasets carry a residual risk of patient re-identification when they are linked with auxiliary information.^61^ The ownership and governance of laboratory, imaging, genomic, and multi-omics data, the security of sensitive clinical information and the handling of data breaches, and the conditions under which data are shared across institutions and national borders all require explicit frameworks.^60^^,^^62^ Accountability and liability become pressing when an AI-supported laboratory conclusion contributes to an incorrect diagnosis or treatment decision, and transparency is complicated by the use of commercial, proprietary, or continuously updated algorithms whose behavior may change over time.^63^ Importantly, conventional ethics approval for the collection of biological samples in routine clinical practice does not automatically cover the additional governance requirements associated with large-scale computational reuse of clinical data, an area that remains debated and subject to interpretation.^62^

Algorithmic bias arising from the underrepresentation of some populations in training data compounds these governance concerns, as does the effect of automation on laboratory workforce. Addressing these challenges will require coordinated efforts across technology developers, regulatory agencies, professional organizations, and healthcare institutions.^57^

These challenges are felt most acutely in low-resource settings, where limited access to curated data, computing infrastructure, and expertise risks widening rather than narrowing existing disparities.^57^ Because the computational infrastructure on which AI depends is required for both discovery research and clinical diagnostics yet is distributed unequally across institutions, regions, and jurisdictions, unequal access may further limit reproducibility and adoption and reinforce inequities in research capacity, diagnostic access, and healthcare delivery.^64^

## 11 Emerging trends and future directions in AI-powered in vitro analysis

Several emerging trends directly affect the constraints described above. Multimodal AI systems, which integrate information from multiple data types such as genomics, medical imaging, wearable and ambient biosensor data, and electronic health records, represent a particularly promising direction for enabling more comprehensive diagnostic assessments.^65^ The convergence of AI with organ-on-a-chip and microphysiological systems may further expand the scope of in vitro analysis by enabling real-time monitoring of complex tissue models that better recapitulate human physiology.^66^ Federated learning, which allows AI models to be trained across multiple institutions without sharing raw data, can broaden access to larger and more diverse training datasets, although the information exchanged during training can itself leak details of institutional data, so privacy-preserving methods remain necessary.^67^ The continued development of explainable AI methods, edge computing for resource-limited settings, and harmonized regulatory frameworks will be essential for realizing the full potential of AI in in vitro analysis. Progress along these lines will determine how quickly the next generation of generalist segmentation algorithms, foundation models, and self-driving laboratory platforms can be translated from research demonstrations into routine practice in diverse laboratory settings.

Methodologically, the field is shifting from task-specific CNNs to transformer-based architectures and large self-supervised foundation models. Vision transformers now underpin general-purpose computational pathology foundation models trained on very large image collections, which can be adapted to diverse diagnostic tasks with comparatively little labeled data,^68^^,^^69^ and transformer architectures are increasingly applied across medical image analysis more broadly.^70^ Together with the federated learning and explainable AI approaches noted above,^56^^,^^59^ these methods may improve generalization and transparency, although they also raise new requirements for computational infrastructure, standardized data, and independent external validation.

A further frontier lies in the application of AI to next-generation in vitro models and New Approach Methodologies (NAMs), which the FDA describes as innovative testing methods encompassing in vitro human-based systems, in silico modeling, and other platforms that can reduce or replace animal testing.^71^ Within this category, complex in vitro models and microphysiological systems,^54^ including organoids, bioprinted constructs, and multicellular co-culture models,^66^^,^^72^ are of relevance to laboratory analysis. ML is increasingly used to construct these systems and to analyze the rich, dynamic image and multi-omics data they generate.^66^^,^^72^ At the same time, increasingly complex models generate increasingly complex datasets, and their biological relevance, reproducibility, and qualification for a defined context of use remain essential yet progressively harder to establish.^54^

## 12 Conclusion

AI has demonstrated substantial potential for improving the efficiency, accuracy, and scalability of in vitro analyses in discovery research and clinical diagnostics. From automated cell segmentation and high-throughput drug screening to real-time QC and biomarker detection, AI-driven approaches have matched expert annotators in benchmark studies while reducing the time required for many laboratory tasks. The growing number of regulatory authorizations for AI-enabled diagnostic devices reflects increasing institutional involvement. The evidence reviewed here nevertheless points to a persistent gap between reported performance and demonstrated clinical value, and four priorities follow: First, validation must move beyond retrospective, single-center benchmarks; pooled accuracy estimates remain compromised by the risk of bias, externally validated models lose performance in new settings, and few devices are evaluated prospectively before their authorization. Second, the field needs multicenter, representative, well-annotated datasets with harmonized metadata and clinically meaningful reference standards, as well as post-deployment monitoring for dataset shift; federated learning eases data-access barriers but does not resolve differences in data quality or measurement platforms. Third, reporting should distinguish technical performance from analytical validity, clinical validity, clinical utility, explainability, accountability, and data-governance frameworks, which must be developed alongside algorithms. Fourth, equitable access to computing infrastructure, curated data, and expertise should be treated as a translational requirement, as unequal access risks widening the disparities AI is proposed to reduce. Progress on these fronts will determine whether multimodal systems, foundation models, and self-driving laboratories move into routine practice and will require coordinated efforts among developers, regulators, laboratory professionals, and healthcare systems.

## CRediT authorship contribution statement

**Zhinya Kawa Othman:** Writing – original draft, Methodology, Conceptualization. **Mohamed Mustaf Ahmed:** Writing – original draft, Data curation. **Rahmatullah Nazari:** Writing – review & editing, Visualization. **Adamu Muhammad Ibrahim:** Writing – review & editing, Visualization.

## Data availability statement

No new data were generated or analyzed in this study.

## Declaration of generative AI and AI-assisted technologies in the manuscript preparation process

The authors acknowledge the use of Paperpal AI (https://paperpal.com/) for its “Language Edit” and “Make Academic” features to improve clarity and readability. This assistance was limited to the linguistic refinement. All analyses and interpretations are solely those of the authors.

## Funding

Not applicable.

## Declaration of competing interest

The authors declare that they have no conflicts of interest.
